# Release of Bioactive Peptides from Whey Protein During In Vitro Digestion and Their Effect on CCK Secretion in Enteroendocrine Cells: An In Silico and In Vitro Approach

**DOI:** 10.3390/molecules31020238

**Published:** 2026-01-10

**Authors:** Anaís Ignot-Gutiérrez, Orlando Arellano-Castillo, Gloricel Serena-Romero, Mayvi Alvarado-Olivarez, Daniel Guajardo-Flores, Armando J. Martínez, Elvia Cruz-Huerta

**Affiliations:** 1 Instituto de Neuroetología, Universidad Veracruzana, Av. Dr. Luis Castelazo Ayala s/n, Industrial Ánimas, Xalapa-Enríquez 91193, Veracruz, Mexico; 2 Centro de Investigaciones Biomédicas, Universidad Veracruzana, Av. Dr. Luis Castelazo Ayala s/n, Industrial Ánimas, Xalapa-Enríquez 91193, Veracruz, Mexico; 3 Escuela de Ingeniería y Ciencias, Tecnológico de Monterrey, Av. Eugenio Garza Sada 2501 Sur, Monterrey 64849, Nuevo León, Mexico; 4 Centro de Investigación y Desarrollo en Alimentos, Universidad Veracruzana, Av. Dr. Luis Castelazo Ayala s/n, Industrial Ánimas, Xalapa-Enríquez 91193, Veracruz, Mexico

**Keywords:** bioactive peptides, cholecystokinin, enteroendocrine cells, protein digestion, satiety hormones, whey protein

## Abstract

During gastrointestinal digestion, dietary proteins are hydrolyzed into peptides and free amino acids that modulate enteroendocrine function and satiety-related hormone secretion along the gut–brain axis, thereby contributing to obesity prevention. We investigated whey protein concentrate (WPC) as a source of bioactive peptides and evaluated the effects of its digests on cholecystokinin (CCK) secretion in STC-1 enteroendocrine cells by integrating the standardized INFOGEST in vitro digestion protocol, peptidomics (LC–MS/MS), and in silico bioactivity prediction. In STC-1 cells, the <3 kDa intestinal peptide fraction exhibited the strongest CCK stimulation, positioning these low-molecular-weight peptides as promising bioactive components for satiety modulation and metabolic health applications. Peptidomic analysis of this fraction identified short sequences derived primarily from β-lactoglobulin (β-La) and α-lactalbumin (α-La), enriched in hydrophobic and aromatic residues, including neuropeptide-like sequences containing the Glu–Asn–Ser–Ala–Glu–Pro–Glu (ENSAEPE) motif of β-La f(108–114). In silico bioactivity profiling with MultiPep predicted antihypertensive, angiotensin-converting enzyme (ACE)–inhibitory, antidiabetic, dipeptidyl peptidase-IV (DPP-IV)–inhibitory, antioxidant, antibacterial, and neuropeptide-like activities. Overall, digestion of WPC released low-molecular-weight peptides and amino acids that enhanced CCK secretion in vitro; these findings support their potential use in nutritional strategies to enhance satiety, modulate appetite and energy intake, and improving cardiometabolic health.

## 1. Introduction

Food intake and energy homeostasis are regulated by a network of central and peripheral signals acting along the bidirectional gut–brain axis. This communication involves neural, endocrine and immune pathways linking the gastrointestinal tract with key brain regions that control appetite, reward, and energy balance [1,2,3]. The gastrointestinal tract is now recognized as a major endocrine organ, secreting numerous peptide hormones that coordinate digestion and satiety [4]. Among gut-derived hormones, cholecystokinin (CCK) is a key mediator of short-term appetite control. CCK is a 27-amino-acid peptide released from enteroendocrine I cells in the small intestine in response to luminal nutrients, particularly proteins and lipids. It regulates antropyloroduodenal motility, stimulates pancreatic enzyme secretion and gallbladder contraction, delays gastric emptying, reduces circulating orexigenic neuropeptide Y (NPY), activates vagal afferent signaling and central CCK receptors to promote satiety [4,5,6]. The growing global burden of overweight and obesity, closely linked to dysregulated appetite and excess energy intake, has intensified interest in nutritional strategies that enhance endogenous satiety signaling via modulation of gut hormones such as CCK and glucagon-like peptide-1 (GLP-1) [3,7,8,9].

Dietary proteins are generally considered the macronutrients with the greatest satiating potential compared with carbohydrates and fats, largely because they stimulate the secretion of anorexigenic intestinal hormones, including CCK, GLP-1, and peptide YY (PYY) [5,6,8,10]. However, their effects on satiety are not uniform, the magnitude and duration of the satiating response depend on the protein source, structure, amino acid composition, and susceptibility to gastrointestinal digestion [8,10,11]. During gastrointestinal digestion, dietary proteins are hydrolyzed by gastric and pancreatic proteases, as well as brush-border peptidases, generating complex mixtures of peptides and free amino acids. These digestion products can interact with nutrient-sensing receptors on enteroendocrine cells, triggering the release of anorexigenic hormones [5,12]. Experimental studies in enteroendocrine cell models such as STC-1 indicate that peptide size and sequence traits, particularly enrichment in hydrophobic and aromatic residues, are key determinants of CCK and GLP-1 stimulatory activity [5,12,13].

Accordingly, the digestion-derived peptide and free amino acid profile is a primary determinant of gut–brain signaling and satiety-related outcomes. Recent reviews emphasize that specific proteins and protein hydrolysates from animal, plant, and alternative sources can promote satiety and contribute to obesity prevention through their effects on gut hormone secretion and appetite regulation [8,10]. Thus, the identification of protein-derived peptides with defined bioactivities relevant to satiety and metabolic control is a strategic objective in the development of functional foods and nutraceuticals aimed at appetite management and obesity prevention [8,10,11].

Whey protein concentrate is a high-quality protein ingredient that provides all essential amino acids and contains major whey proteins such as β-lactoglobulin, α-lactalbumin, serum albumin, immunoglobulins, and lactoferrin [14,15]. Whey proteins are rapidly digested and absorbed and have been associated with reduced short-term food intake and favorable postprandial metabolic responses [8,14]. Beyond their nutritional value, whey-derived peptides exhibit a broad spectrum of biological activities, including antihypertensive, ACE-inhibitory, antidiabetic, anti-inflammatory, antioxidant, antimicrobial, and immunomodulatory effects [16,17,18,19].

Recent bibliometric and functional analyses suggest that whey peptides are a rich source of multifunctional bioactive sequences that have the potential to promote cardiometabolic health, gut homeostasis, and may have neuroprotective effects [17,18,20]. From an obesity perspective, whey proteins reduce food intake in the short term, probably due to their rapid digestion and branched-chain amino acid content [8,21]. At the same time, they supply high-quality amino acids and generate peptide pools capable of influencing gut–brain communication, satiety, and metabolic regulation [8,17,22].

The increasing accessibility of curated bioactive peptide databases and advanced bioinformatics tools has expedited the identification of food-derived bioactive peptides. In silico approaches, including simulated proteolysis, sequence-based bioactivity prediction and ADME/bioavailability modeling allow rapid screening of large peptide datasets and prioritization of candidates for experimental validation, thereby complementing conventional in vitro and in vivo assays [23,24]. Studies applying these strategies to dairy and other food proteins have demonstrated the effectiveness of combining in vitro digestion with in silico profiling to identify peptides with antihypertensive, antidiabetic, anti-inflammatory and antioxidant potential [16,25,26].

We hypothesize that bioactive peptides with a molecular weight below 3 kDa, released during gastrointestinal digestion of WPC, stimulate CCK secretion in intestinal STC-1 enteroendocrine cells, thereby contributing to enhanced satiety and reduced food intake. Therefore, this study aimed to investigate the effects of WPC digests and peptide fractions with molecular weights lower or greater than 3 kDa on CCK secretion in STC-1 cells and to identify peptide sequences released during in vitro intestinal digestion of WPC that may underlie this secretagogue effect. By integrating in vitro gastrointestinal digestion, peptide identification by HPLC–MS/MS, and in silico bioactivity profiling, this work aims to identify multifunctional whey-derived peptides capable of modulating the gut–brain axis, enhancing satiety, and potentially supporting strategies for appetite control and obesity prevention.

## 2. Results

### 2.1. Effect of In Vitro Digestion on WPC Proteins and Free Amino Acid Release

#### 2.1.1. In Vitro Digestibility

**Figure 1:**
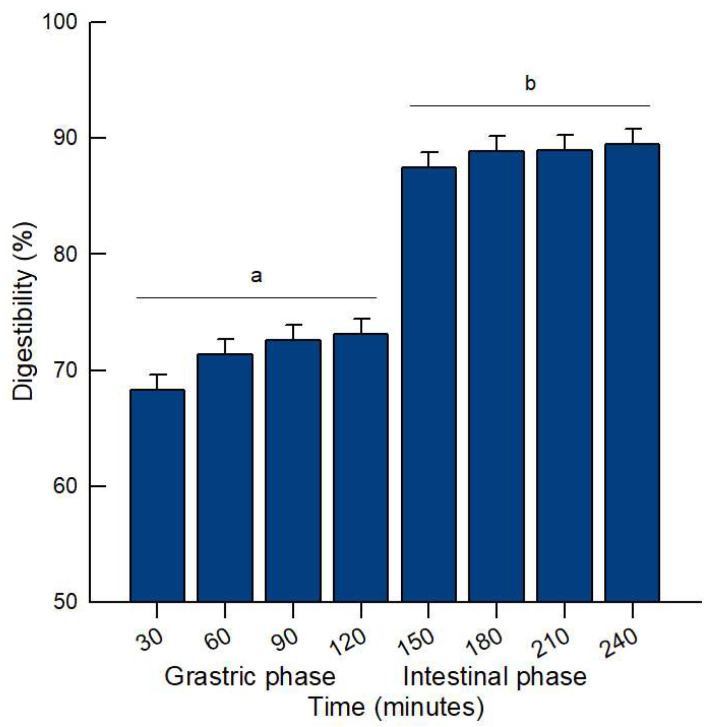
Digestibility (mean ± SEM) of WPC digests during the gastric and intestinal phases at different times 30, 60, 90, 120, 150, 180, 210, and 240 minutes (min). Statistical analysis was performed by one–way ANOVA (df = 7, F = 51.35, *p* < 0.001). Means not sharing the same letters indicate differences in percentages according to Tukey’s method (α = 0.05).

Among milk proteins, whey proteins exhibit substantially higher digestibility than caseins, resulting in greater amino acid availability and, consequently, higher nutritional value. In this study, the initial protein content of the WPC was 79.8%. From the earliest stages of in vitro gastrointestinal digestion, digestibility increased steadily due to enzymatic hydrolysis and the structural characteristics of the proteins. During the gastric phase, a rapid rise in WPC digestibility was observed: after 30 min of gastric digestion, digestibility reached 68.31% (Figure 1), increasing slightly to 73.15% by the end of this phase. In the first 30 min of the intestinal phase (150 min total digestion time), digestibility further increased to 87.49%, and by the end of the intestinal phase (240 min), it reached 89.51%.

Consistent with these changes, the protein content decreased after each phase of gastrointestinal digestion, reflecting the progressive release of peptide sequences and free amino acids because of enzymatic action. A substantial proportion of the proteins was hydrolyzed during the gastric phase by pepsin, leading to a marked reduction in protein content, which was further enhanced after digestion with pancreatin. These results support the classification of whey proteins as fast-digesting [27,28]. Whey proteins are considered fast due to their high rate of amino acid absorption and rapid action on endogenous protein synthesis. After ingestion, whey proteins reach the jejunum quickly in the form of polypeptides and are gradually digested in the small intestine, in contrast to other proteins [29,30].

#### 2.1.2. SDS–PAGE

**Figure 2:**
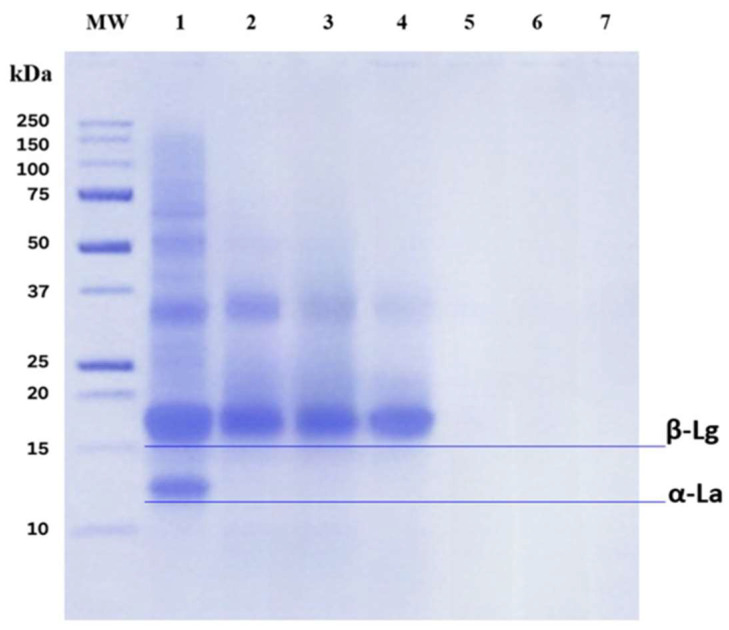
SDS–PAGE of WPC during simulated gastrointestinal digestion under reducing conditions. Lane assignments: MW, molecular weight marker; 1, WPC (undigested); 2, G30 (gastric, 30 min); 3, G60 (gastric, 60 min); 4, G120 (gastric, 120 min); 5, I30 (intestinal, 30 min); 6, I60 (intestinal, 60 min); 7, I120 (intestinal, 120 min).

Electrophoresis displayed a characteristic whey protein pattern with bands between 14 and 80 kDa (Figure 2). The band at approximately 66 kDa most likely corresponds to bovine serum albumin (BSA), the band at 18 kDa to β-lactoglobulin (β-Lg), and the band at 14 kDa to α-lactalbumin (α-La) [27,31]. The intensity of bands corresponding to higher-molecular-mass proteins decreased progressively with digestion time, consistent with proteolysis by gastrointestinal enzymes. During the gastric phase, BSA hydrolysis was evident within the first 60 min, with the appearance of lower-molecular-weight fragments (37 kDa). By contrast, β-Lg was largely resistant to pepsin but was rapidly degraded by pancreatin at the onset of the intestinal phase, in line with the known resistance of β-Lg to pepsin both in vitro and in vivo [32]. This resistance is attributed to its compact three-dimensional structure and disulfide bonds, although pH and protein–enzyme interactions can further modulate susceptibility [29,33,34].

Overall, the progressive fading of intact-protein bands and the emergence of low-molecular-weight material suggest the formation of peptides below the gel’s resolving range during simulated gastrointestinal digestion. Thus, digestion leads to extensive hydrolysis and the release of peptides and free amino acids, whose functional effects will depend on sequence and physicochemical properties [34]. Consistent with these results, Tulipano et al. [29] reported that residual intact or partially hydrolyzed whey proteins, such as β-Lg may interact with the intestinal mucosa and facilitate the delivery of longer peptides capable of acting on enteroendocrine cells.

#### 2.1.3. Free Amino Acids in Digests

**Figure 3:**
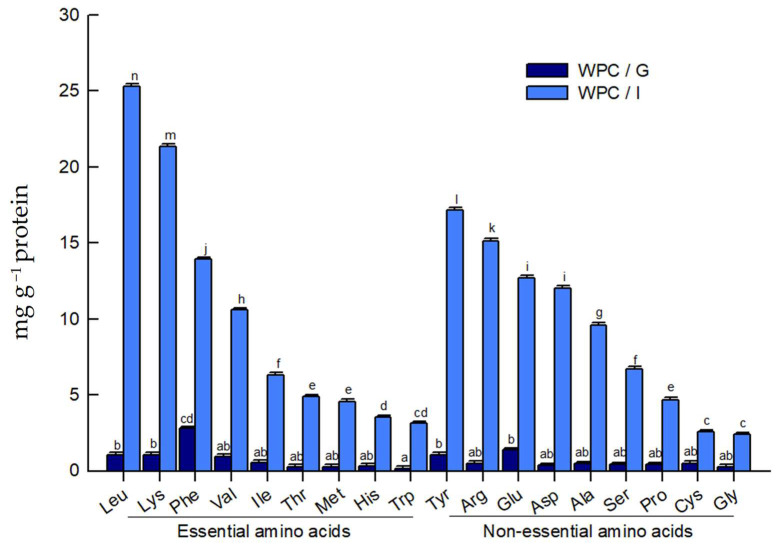
Free amino acid profile in whey protein concentrate (WPC) following simulated gastrointestinal digestion (mg g^−1^ protein; mean ± SEM; *n* = 3). Results are shown for gastric (WPC/G, dark blue) and intestinal (WPC/I, light blue) digests, grouped by essential (left) and non-essential (right) amino acids. Data were analyzed by two-way ANOVA (see Supplementary Table S1). Means not sharing the same letters indicate differences according to Tukey’s method (α = 0.05), comparing all pairs of means.

The determination of free amino acids provides an indirect measure of the degree of hydrolysis of WPC. The content of free amino acids for gastric and intestinal digestates is shown in Figure 3. After the intestinal phase, total free amino acids increased markedly, rising from 17.377 ± 0.04 mg g^−1^ protein after gastric digestion to 232.577 ± 0.04 mg g^−1^ protein at the end of gastrointestinal digestion. This increase is attributed to the action of digestive enzymes that cleave whey proteins at multiple sites, releasing peptides and free amino acids with potential biological activities, including the stimulation of enteroendocrine hormone secretion, such as CCK and GLP-1 [34]. These results are in line with recent in vitro and in vivo studies showing that gastrointestinal digestion of whey proteins generates substantial amounts of free amino acids and small peptides that can act as nutrient signals for satiety-related hormones [34,35,36].

Among the essential amino acids, leucine and lysine were the most abundant (25.293 and 21.334 ± 0.15 mg g^−1^ protein, respectively), followed by phenylalanine, valine, and isoleucine (13.917, 10.573, and 6.298 ± 0.15 mg g^−1^ protein, respectively). Among the nonessential amino acids, tyrosine, arginine, and glutamic acid showed the highest levels (17.179, 15.134, and 12.685 ± 0.15 mg g^−1^ protein, respectively), followed by aspartic acid and alanine (12.036 and 9.602 ± 0.15 mg g^−1^ protein, respectively). Hydrophobic residues predominated overall; aromatic amino acids were relatively enriched in the gastric digest, whereas branched-chain amino acids (BCAAs) were enriched in the intestinal digest. This free amino acid profile supports the well-recognized high digestibility of whey proteins and their favorable supply of both essential and nonessential amino acids [14,33,34,37]. Notably, aromatic amino acids increased after gastrointestinal digestion, particularly tyrosine, histidine, phenylalanine, and tryptophan; the latter two have been reported to stimulate CCK via the calcium-sensing receptor (CaSR) [35,38].

Intestinal whey protein digests showed increased release of branched-chain amino acids (BCAAs; leucine, valine, and isoleucine), which have been implicated in activating nutrient-sensing receptors and secretagogue pathways [39,40], along with higher levels of lysine, methionine, phenylalanine, proline, and tyrosine [36,37]. Human intervention studies have reported that postprandial rises in circulating BCAAs and aromatic amino acids after protein-rich meals are associated with increased secretion of CCK, GLP-1 and other appetite-regulating hormones, supporting a contributory role of these amino acids as metabolic signals in gut–brain communication [21,37]. In an in vivo study, Elovaris et al. [36] reported positive associations between postprandial increases in appetite-regulating hormones, including CCK, and circulating amino acids such as BCAAs, lysine, methionine, aspartic acid, tryptophan, and tyrosine, all of which were abundant in our intestinal digests. Taken together, these findings suggest that the satiogenic effects of dietary proteins reflect their distinct amino acid compositions and the peptide/free amino acid mixtures generated during digestion.

### 2.2. CCK Secretion in STC–1 Cells Induced by Whey Protein Digests

Before the CCK secretion assays, cell viability was assessed to determine the cytotoxic effect of the WPC and the >3 kDa and <3 kDa fractions of gastric and intestinal digests at concentrations of 1 and 4 mg mL^−1^ (Supplementary Materials Table S2). All treated concentrations maintained viability above 80%, indicating the nontoxic nature of the samples at these doses. These results were used to establish the concentrations range selected for the subsequent assays evaluating CCK secretion.

**Figure 4:**
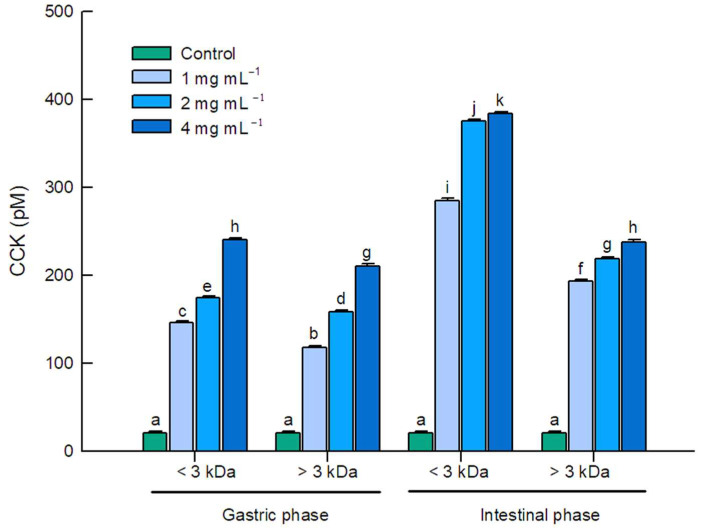
CCK secretion by STC-1 cells in response to WPC digests and their molecular mass fractions. STC-1 cells were incubated with gastric or intestinal WPC digests fractionated into <3 kDa and >3 kDa fractions at 1, 2, or 4 mg mL^−1^; cells incubated with HEPES buffer in the absence of digests served as the control. CCK concentrations in culture supernatants are reported as pM (mean ± SEM; *n* = 3 independent experiments, each performed in technical duplicate). Statistical analyses were conducted using three-way ANOVA (Supplementary Tables S3 and S4). Means that do not share the same letters indicate differences according to Tukey’s method (α = 0.05), comparing all pairs of means. Absolute values reflect in vitro release from STC-1 cultures and are not intended to represent systemic concentrations.

CCK secretion in STC-1 cells after 1 h of incubation with the <3 kDa and >3 kDa fractions of the gastric and intestinal digests of WPC at three concentrations (1, 2, and 4 mg mL^−1^) was quantified by CCK-like immunoreactivity in the cell culture supernatant (Figure 4). Importantly, the absolute CCK concentrations reported here reflect accumulation in the culture medium of murine STC-1 cells under in vitro conditions and should not be interpreted as surrogates for circulating postprandial CCK levels in humans. The results showed an increase in CCK secretion for all fractions in a dose-dependent manner compared with the control, as supported by the interactions identified by the applied statistical model (Supplementary Materials Tables S3 and S4).

At the three concentrations tested (1, 2, and 4 mg mL^−1^), the <3 kDa fraction of the gastric digest (146,351; 174,850, and 240,198 ± 2215 pM, respectively) elicited greater CCK secretion compared with the >3 kDa fraction (118,158; 158,628; and 210,766 ± 2215 pM, respectively), reaching up to 11-fold above the control. Secretion was even higher for the intestinal digest fractions at the same concentrations. The <3 kDa intestinal fraction yielded CCK concentrations of 285,171; 375,428; and 383,433 ± 2215 pM, respectively, corresponding to as much as 18-fold above the control, whereas the >3 kDa intestinal fraction showed concentrations of 193,138; 218,985; and 238,154 ± 2215 pM, respectively. In addition to the clear dose-dependent trend, these results indicate that the <3 kDa fractions consistently stimulated greater CCK secretion than the >3 kDa fractions in both phases of digestion.

Notably, the intestinal <3 kDa fraction contains peptides as well as free amino acids, both of which can stimulate CCK secretion via nutrient-sensing receptors [12,39]. Accordingly, its superior secretagogue activity likely reflects the combined or even synergist contribution of digestion-derived peptides and coexisting free amino acids [5,36]. To accurately delimit the relative contribution of each component, future studies should directly compare peptide fractions with compositionally matched free amino acid mixtures at equal concentration, evaluating in parallel CCK secretion and satiety signaling indicators.

Overall, these findings indicate that low-molecular-weight digestion products present in the ultrafiltration-derived permeate fractions enhance CCK secretion, particularly in the intestinal digest. Peptide length, sequence context, and terminal-residue chemistry likely modulate secretagogue potency. Short peptides and free amino acids can activate G-protein-coupled receptors (GPCRs) and the calcium-sensing receptor (CaSR), thereby promoting CCK release [12,38,39]. Consistent with our observations, previous reports have shown that low-molecular-weight peptides from dietary proteins more potently stimulate CCK release in STC-1 cells than larger peptides or intact proteins [22,40,41], and that peptide sequences generated during protein hydrolysis elicit stronger CCK synthesis and release than equimolar mixtures of free amino acids [5,36,39].

Several work on milk- and cereal-derived peptides has further underscored that short (≈4–11-residue), hydrophobic, and aromatic-residue–enriched sequences are particularly effective at stimulating CCK and GLP-1 secretion via CaSR- and GPCR-dependent pathways in STC-1 cells and in vivo models [8,10,12,39,42]. These findings, together with recent evidence demonstrating the role of low-molecular-weight peptides from dairy and other food proteins in enteroendocrine hormone secretion, support the importance of the peptide profile generated during gastrointestinal digestion for modulating CCK-mediated satiety signaling.

### 2.3. Peptides Released During In Vitro Gastrointestinal Digestion of WPC

**Figure 5:**
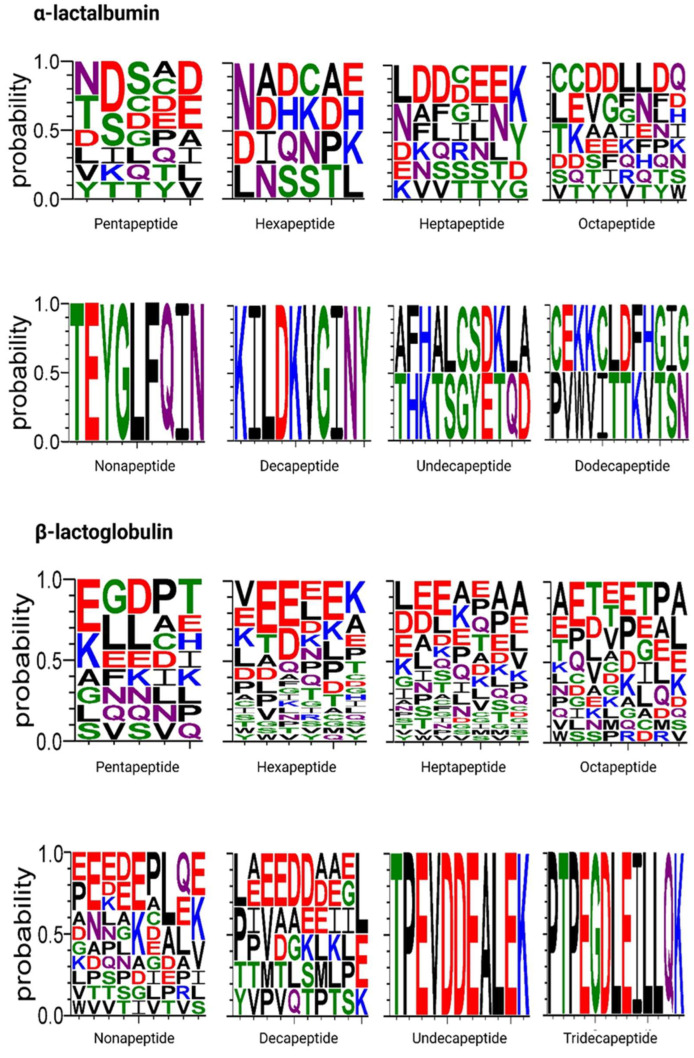
Sequence of peptides released from whey protein concentrate (α–Lactalbumin and β–Lactoglobulin) during simulated gastrointestinal digestion. Logos depict amino-acid frequency (y-axis, probability) for peptides identified by HPLC–MS/MS in the <3-kDa fraction collected after 120 min of the intestinal phase. Panels show peptides grouped by length and source protein: α-lactalbumin (pentapeptides to dodecapeptides, top two rows) and β-lactoglobulin (pentapeptides to tridecapeptides, bottom two rows). Sequences were aligned at the N-terminus; *n* = 37 α-lactalbumin peptides and *n* = 93 β-lactoglobulin peptides. Logos were generated with WebLogo 2.8.2. Color code: acidic (D/E, red), basic (H/K/R, blue), neutral polar (N/Q, purple), polar (C/G/S/T/Y, green), and hydrophobic/aromatic (A/F/I/L/M/P/V/W, black).

Peptides in the <3 kDa fraction of WPC digests after 120 min of the intestinal phase were identified by HPLC–MS/MS. A total of 130 peptides were identified, with 37 peptide sequences corresponding to α-lactalbumin and 93 to β-lactoglobulin (Supplementary Materials Table S5 and Figure S1). The α-lactalbumin peptide sequences were identified in several regions, predominantly f(21–56) and f(74–104) Most α-lactalbumin peptide sequences contained 5 or 8 amino acids (10 sequences), followed by 7 and 6 amino acids (7 and 4 sequences, respectively). Additional peptides of 9–12 amino acids were identified in lower numbers. The β-lactoglobulin peptide sequences were primarily found in regions f(58–76) and f(124–151). Peptide sequences of 7 and 6 amino acids (27 and 24 sequences, respectively) were mainly identified, followed by sequences composed of 8, 9, and 5 amino acids (14, 11, and 9 sequences, respectively), with additional peptides of 10, 11, and 13 amino acids detected in smaller numbers (Figure 5).

These results are consistent with previous in vitro and in vivo peptidomics studies, in which β-lactoglobulin peptides are mainly detected in the central and C-terminal regions of the protein (approximately f(40–70) and f(120–150)), and α-lactalbumin peptides cluster in specific domains rather than being evenly distributed along the sequence [32,34,43]. Peptide lengths observed here (primarily 5–13 residues) are within the range typically released from whey proteins during gastrointestinal digestion and are comparable to those reported in physiological human digestion [43,44,45].

The peptides identified under our simulated conditions appear representative of those generated in vivo during the digestion of whey protein ingredients, supporting the physiological relevance of the present in vitro model [32,34,43]. To relate the sequences to known functions, all <3 kDa intestinal peptides were queried against the Milk Bioactive Peptide Database (MBPDB) (Table 1). Ten of the 130 peptides showed high sequence identity (including several exact matches, 100% identity) to previously reported bioactive peptides. MBPDB has recently been expanded and curated as a comprehensive resource for milk protein–derived bioactive peptides, integrating structural, functional, and bioactivity annotations, which strengthens the interpretation of the present matches [46,47].

Low-molecular-mass peptides can interact with cellular receptors and elicit biological responses [12,34]. In particular, peptides of ≥5 amino acid residues generated during gastrointestinal digestion have been implicated in CCK secretion in enteroendocrine STC-1 cells [12,39]. Recent studies show that short peptides 4–11 residues enriched in hydrophobic and aromatic residues, effectively stimulate CCK and GLP-1 secretion through CaSR- and GPCR-dependent pathways in STC-1 cells and in vivo models [12,42]. Several sequences identified in β-lactoglobulin and α-lactalbumin in this study contain terminal residues such as Phe, Val, Trp, and Lys, which have been associated with the release of satiety-related hormones (CCK, GLP-1) [35] and aromatic residues are frequently enriched in CCK-stimulatory peptides [42].

Functionally annotated matches included, for α-lactalbumin, the peptides DKVGINYW and LAHKAL, both previously reported as angiotensin-converting enzyme (ACE) inhibitors [48]. Among the peptides identified from β-lactoglobulin, two sequences (LIVTQTMK and LIVTQTMKGL) were highly similar to LIVTQTMKG, known as lacto-ghrestatin, which was identified through bioinformatic analysis [49] and has been shown to have an inhibitory effect on ghrelin secretion in the MGN3-1 cell line [50]. Moreover, peptide sequences analogous to previously reported bioactive peptides were observed, including TPEVDDEALEK, described as having antimicrobial activity and dipeptidyl peptidase-IV (DPP-IV) inhibitory effects, and DAQSAPLRVY, reported as an ACE inhibitor [47]. The sequence LKPTPEGDL has also been described as a DPP-IV inhibitor [46,47]. These findings are in line with recent reviews highlighting that whey-derived peptides frequently display multiple overlapping bioactivities, including ACE inhibition, DPP-IV inhibition, antioxidant and antimicrobial effects, and may contribute to the modulation of cardiometabolic and inflammatory pathways [17,18,20,29]. However, it is noteworthy that the peptide ALPMH, derived from β-lactoglobulin and previously reported to stimulate CCK secretion [22], was not identified under our experimental conditions.

Therefore, the CCK secretagogue effect demonstrated by the intestinal digest is likely mediated by other peptide sequences with different but complementary structural determinants. Peptides released during gastrointestinal digestion of food proteins can stimulate CCK secretion more strongly than individual amino acids [39,40,44]. Peptide length and amino-acid composition, including the distribution of hydrophobic, aromatic, and charged side chains, are key determinants of activity in enteroendocrine cells [22,29,39,44]. Furthermore, recent peptidomic analyses suggest that bioactivity may depend not only on linear sequence motifs but also on the spatial arrangement of critical residues imposed by the three-dimensional conformation of the peptide, which can modulate receptor binding and downstream signaling [16,23,24].

### 2.4. In Silico Bioactivity Profiling of Whey-Derived Peptides

**Figure 6:**
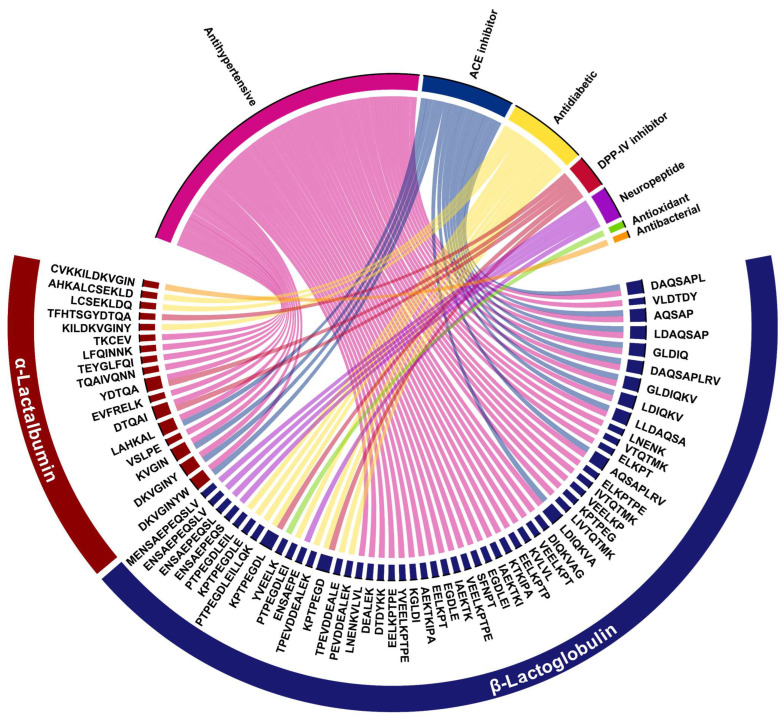
Chord diagram summarizing the in silico bioactivity profiling of 70 whey-derived peptides (MultiPep score ≥ 0.70) identified by HPLC–MS/MS in the <3 kDa intestinal fraction after simulated gastrointestinal digestion of whey protein concentrate. The peptides were 5–13 residues in length and included 17 sequences from α-lactalbumin and 53 from β-lactoglobulin. In the plot, sectors of the outer ring represent the protein of origin of each peptide: α-lactalbumin (red) and β-lactoglobulin (blue). Sectors of the inner ring correspond to predicted biological functions: antihypertensive (pink), angiotensin-converting enzyme (ACE) inhibitor (dark blue), antidiabetic (yellow), dipeptidyl peptidase-IV (DPP-IV) inhibitor (red), neuropeptide (purple), antioxidant (green), and antibacterial (orange). Color-coded chords link individual peptide sequences to one or more predicted activities.

Of the 130 peptides identified by LC–MS/MS in the <3 kDa intestinal fraction, 70 showed MultiPep prediction scores ≥ 0.70 and were therefore selected for in silico bioactivity profiling (Supplementary Materials Table S6). As illustrated in Figure 6, the chord diagram depicts the relationships between these 70 candidate bioactive peptides and seven predicted bioactivity classes, summarizing the distribution of antihypertensive, ACE-inhibitory, antidiabetic, DPP-IV-inhibitory, neuropeptide, antioxidant, and antibacterial candidates. In this diagram, each peptide sequence is connected to its predicted biological activity by color-coded chords; the width of each activity sector reflects the total number of peptides associated with that function, whereas the thickness of individual chords represents the co-occurrence of multiple predicted activities for a given peptide. Of the peptides shown, 17 originated from α-lactalbumin and 53 from β-lactoglobulin. The identified peptides ranged from 5 to 13 amino acid residues, a size range consistent with the low-molecular-weight peptides typically released from whey proteins during gastrointestinal digestion [27,32,43,51].

The bioactivity most frequently predicted was antihypertensive activity (50 peptide sequences), followed by angiotensin-converting enzyme (ACE) inhibitory predictions (14 peptide sequences) and antidiabetic predictions (11 peptide sequences). Additionally, five peptide sequences were predicted to be potential dipeptidyl peptidase-IV (DPP-IV) inhibitors. The chord diagram also highlights extensive functional overlap, as many peptides are simultaneously assigned to more than one bioactivity class (for example, antihypertensive/ACE inhibitory and antihypertensive/antidiabetic), in agreement with the notion that whey-derived peptides often display pleiotropic biological effects [29,47].

Among the α-lactalbumin–derived peptides, DKVGINYW, DKVGINY, KVGIN, and LAHKAL showed the highest probabilities for antihypertensive activity and ACE-inhibitory, with values approaching 1.00. A second cluster, including AHKALCSEKLD, LCSEKLDQ, KILDKVGINY, NNDSTE, and KALCSEK, displayed elevated antidiabetic probabilities (0.80–0.91) with concurrent, moderate DPP-IV inhibition, suggesting potential effects on glycemic control. In parallel, short peptides such as YDTQA, DTQAI, TSGYD and the extended TFHTSGYDTQA reached high DPP-IV–inhibitory probabilities (up to 0.97), often co-occurring with predicted antihypertensive and antioxidant probabilities such as VSLPE and DDQNPH.

In β-lactoglobulin, MultiPep identified 53 peptides with at least one predicted bioactivity (probability ≥ 0.70), delineating a dense cluster of sequences with cardiometabolic relevance. Peptides harboring the Asp–Ala–Gln–Ser–Ala–Pro (DAQSAP) motif such as AQSAP, LDAQSAP, DAQSAPL, DAQSAPLRV, together with fragments centered on GLDIQ/DIQKV (GLDIQ, GLDIQKV, LDIQKV) and related segments (LLDAQSA, LNENK, VTQTMK) exhibited the highest predicted antihypertensive and ACE-inhibitory probabilities, in several cases approaching 1.00. This pattern supports the notion that DAQSAP- and GLDIQ-containing regions constitute privileged structural motifs for ACE inhibition and blood-pressure regulation within the β-lactoglobulin sequence.

A second prominent cluster comprised Lys–Pro–Thr–Pro–Glu–Gly–Asp (KPTPEGD)–based peptides (KPTPEGDL, KPTPEGDLE, PTPEGDLEI, PTPEGDLEIL) and the Glu-rich fragment TPEVDDEALEK, which showed high predicted antidiabetic and DPP-IV–inhibitory probabilities while retaining moderate antihypertensive potential. Additional sequences such as YVEELK, EGDLE, DTDYKK, VEELKP and ELKPT, displayed elevated predicted antioxidant probabilities, suggesting complementary protection against oxidative stress.

In addition, MultiPep flagged a small cluster of five ENSAEPE-based peptides (ENSAEPEQSL, ENSAEPEQSLV, MENSAEPEQSLV, ENSAEPEQS, and ENSAEPE) with high neuropeptide prediction scores (0.71–0.98), with ENSAEPEQSL and ENSAEPEQSLV exhibiting the highest values. All of these sequences share the acidic ENSAEPE motif corresponding to β-La residues 108–114, which confers a net negative charge at physiological pH and may modulate their interactions with peptide receptors and other signaling targets [38]. Their neuropeptide-like prediction signatures are consistent with a potential role in neuromodulatory or hormone-like signaling along the gut–brain axis and warrant targeted evaluation in vitro and in vivo.

Importantly, several peptides in our dataset combine structural traits that have recently been associated with CCK secretagogue activity in enteroendocrine STC-1 cells, such as short length (approximately 4–11 residues), enrichment in hydrophobic and aromatic residues such as Leu, Phe, Trp, and the presence of acidic or γ-glutamyl-containing motifs at the N- or C-terminus [7,38,40]. These features are consistent with activation of CaSR- and G-protein-coupled receptor-dependent mechanisms in STC-1 cells and in vivo models [20,38,45].

For instance, Santos-Hernández et al. [44] identified several milk- and egg-derived peptides that stimulate CCK and GLP-1 secretion in STC-1 cells, underscoring the critical role of specific sequence motifs and residues in receptor activation. Similarly, Vivanco-Maroto et al. [45] recently reported the β-lactoglobulin fragment ^126^TPEVDDEALEKFD^138^ as a potent CCK-inducing peptide in STC-1 enteroendocrine cells, using jejunal contents from human volunteers and simulated gastrointestinal digests of casein and whey proteins. This Glu-rich sequence, characterized by multiple acidic residues, was reported to be particularly effective at stimulating CCK secretion, supporting the concept that negatively charged, glutamate-containing peptides act as potent CCK secretagogues. Notably, our peptidomic analysis also detected related β-lactoglobulin–derived sequences such as TPEVDDEALEK in the <3 kDa intestinal fraction. More broadly, recent reviews on satiety-related peptides and whey-derived bioactive peptides suggest that low-molecular-weight sequences, often bearing hydrophobic and aromatic residues at their termini, are among the most plausible modulators of gut hormones involved in appetite regulation and metabolic control [12,22].

Our in silico profile is consistent with recent comprehensive analyses of milk-derived bioactive peptides. These analyses report that ACE-inhibitory, antihypertensive, antimicrobial, antioxidant, and DPP-IV-inhibitory activities are among the most frequently annotated functions in curated databases and systematic reviews [46,47]. In this context, the distribution of predicted activities in our WPC digest (with a predominance of antihypertensive/ACE-inhibitory and antidiabetic/DPP-IV-inhibitory peptides) mirrors the functional landscape described for milk peptides at large. Furthermore, our use of MultiPep to predict multifunctional peptides aligns with current bioinformatics approaches that integrate in silico digestion, activity prediction, and database mining to prioritize candidate bioactive peptides for experimental validation [47,49].

These approaches are increasingly recognized as cost-effective strategies for narrowing the peptide sequence space generated by gastrointestinal digestion. However, they do not constitute evidence of physiological efficacy. In vitro and in vivo studies are still necessary to verify bioactivity, establish bioavailability, and delineate receptor-specific mechanisms. Thus, although MultiPep does not explicitly classify CCK-stimulatory peptides, the structural features of a subset of the antihypertensive, ACE-inhibitory, and antidiabetic sequences identified here, together with current evidence on food-derived CCK-releasing peptides, support the hypothesis that these whey-derived peptides are plausible candidates to underlie the CCK-releasing effects observed for the <3 kDa intestinal WPC fraction.

## 3. Materials and Methods

### 3.1. Materials

Whey protein concentrate was obtained from Clarity Proteins^®^ (León, Guanajuato, Mexico). Pepsin from porcine gastric mucosa (P7012), pancreatin from porcine pancreas (P7545), bovine bile (B3883), 4–(2–aminoethyl) benzenesulfonyl fluoride (Pefabloc^®^ SC, 76307) were purchased from Sigma-Aldrich/Merck (St. Louis, MO, USA) for used in the in vitro digestion experiments. Dulbecco’s Modified Eagle Medium (DMEM) was obtained from Thermo Fischer Scientific (Waltham, MA, USA). Fetal bovine serum (FBS), phosphate–buffered saline pH 7.4 (PBS), trypsin–EDTA 0.25%, penicillin (10,000 U/mL), and streptomycin (10,000 μg/mL) were acquired from Gibco (Grand Island, NY, USA). The CellTiter 96 Aqueous One Solution Cell Proliferation Assay was obtained from Promega Corporation (Madison, WI, USA). All other reagents used were of HPLC grade and were obtained from Sigma-Aldrich/Merck. The murine enteroendocrine cell line STC-1 (ATCC CRL-3254) was obtained from the American Type Culture Collection (Manassas, VA, USA).

### 3.2. In Vitro Gastrointestinal Digestion of Whey Protein Concentrate

The in vitro gastrointestinal digestion of WPC was performed according to the standardized INFOGEST protocol [52], which includes sequential oral, gastric, and intestinal phases. Briefly, WPC (10 g) was diluted in simulated salivary fluid (1:1, *w*/*v*) at pH 7.0 containing CaCl_2_, and the mixture was incubated at 37 °C with constant agitation (150 rpm) for 2 min. Subsequently, the gastric phase was initiated by diluting the oral bolus with simulated gastric fluid (1:1, *w*/*v*) containing porcine pepsin (2000 U/mL) and CaCl_2_. The pH was adjusted to 3.0 with 5 M HCl, and samples were incubated at 37 °C under agitation (150 rpm) for 2 h. Aliquots were collected at the end of each phase and stored at −80 °C for subsequent analysis.

For the intestinal phase, the gastric chyme was mixed 1:1 (*w*/*v*) with simulated intestinal fluid supplemented with bile salts (10 mM) and pancreatin (100 U/mL). The pH was adjusted to 7.0 with NaHCO_3_, and digestion proceeded at 37 °C with shaking (150 rpm) for 2 h. Digestion was terminated by adding the protease inhibitor Pefabloc^®^ to a final concentration of 5 mM, after which samples were stored at −80 °C until analysis. The digestions were performed in triplicate. A digestion blank was prepared under identical conditions, using the same enzyme concentrations but without the protein substrate.

### 3.3. Fractionation of Digests by Ultrafiltration

Gastric and intestinal digests were fractionated by ultrafiltration using centrifugal filter units with a 3 kDa molecular weight cut-off (MWCO) membrane (Vivaspin 2, Cytiva Life Sciences, Marlborough, MA, USA). Samples were centrifuged at 4000× *g* for 40 min at 4 °C to obtain permeate (<3 kDa) and retentate (>3 kDa) fractions. Both fractions (<3 kDa and >3 kDa) were collected separately, freeze-dried, and stored at −80 °C until analysis.

### 3.4. In Vitro Protein Digestibility

Protein samples subjected to in vitro digestion were collected at different time intervals (0, 30, 60, 90, 120, 150, 180, 210, and 240 min) during the gastric and intestinal phases. Collected samples were centrifuged at 5000× *g* for 30 min at 4 °C, and the resulting supernatants were lyophilized and stored at −80 °C until further analysis. Protein concentration was determined using the Bradford assay [53], with bovine serum albumin (BSA) as the standard. In vitro protein digestibility was calculated following the methodology described by Najdi Hejazi et al. [54], using Equation (1):


(1) $$Digestibility\left(\%\right)=\frac{\left(BP-AP\right)}{BP}\times 100$$

BP: protein content before in vitro digestion.

AP: protein content after in vitro digestion.

### 3.5. Sodium Dodecyl Sulfate–Polyacrylamide Gel Electrophoresis (SDS–PAGE)

Protein samples were prepared to a final concentration of mg mL^−1^ in sample buffer containing 0.05 M Tris–HCl (pH 6.8), 1.6% (*w*/*v*) sodium dodecyl sulfate (SDS), 8% (*v*/*v*) glycerol, 2% (*v*/*v*) β-mercaptoethanol, and 0.002% (*w*/*v*) bromophenol blue. Samples were heated at 95 °C for 5 min before loading. Electrophoresis was performed on polyacrylamide gels under denaturing conditions at 150 V for 60 min using XT MES running buffer (Bio-Rad Laboratories, Hercules, CA, USA) and a PowerPac™ Basic power supply (Bio-Rad). After electrophoresis, gels were carefully removed, rinsed twice with distilled water, and stained for 1 h with a Coomassie Brilliant Blue staining solution (50% water, 40% methanol, 10% Coomassie Blue). Destaining was performed overnight in a solution containing 70% water, 20% methanol, and 10% glacial acetic acid with gentle agitation. Protein bands were visualized and analyzed using GelAnalyzer 19.1 software (www.gelanalyzer.com; Lazar I. Jr. and Lazar I.). Experiments were performed in triplicate, followed by technical duplicates.

### 3.6. Free Amino Acid Analysis

Free amino acids in the digested samples were determined according to the method described by Serena-Romero et al. [55]. Lyophilized digestates (0.2 g) were dissolved in 4 mL of 5% (*w*/*v*) 5-sulfosalicylic acid and stirred for 1 min. Subsequently, the mixtures were then stored at 4 °C for 1 h and centrifuged at 15,000× *g* for 15 min at 4 °C. The pH of the resulting supernatant was adjusted to 2.2 with 0.3 M NaOH and filtered through a 0.45 µm membrane filter. Free amino acids were quantified by cation-exchange chromatography with post-column ninhydrin derivatization. Detection was performed colorimetrically at 440 and 570 nm using an amino acid analyzer (JLC-500/V AminoTac™, JEOL Ltd., Tokyo, Japan).

Tryptophan determination. Because tryptophan (Trp) can be underestimated with ninhydrin-based detection, free Trp was quantified separately by RP-HPLC with fluorescence detection (λ_ex 280 nm, λ_em 350 nm), as described by Çevikkalp et al. [56].

### 3.7. Cell Assays

The murine STC–1 cell line, supplied by ATCC (ATCC CRL-3254, Manassas, VA, USA), is widely used as an in vitro model to assess foods or compounds that modulate gastrointestinal hormone secretion, such as GLP–1 and PYY. STC–1 cells were cultured in Dulbecco’s modified Eagle’s medium (DMEM) supplemented with 10% (*v*/*v*) fetal bovine serum (FBS, BioWest, Nuaillé, France) and 1% (*v*/*v*) penicillin, streptomycin solution (BioWest) at 37 °C in a humidified atmosphere containing 5% CO_2_ and 95% relative humidity. When cultures reached 80% confluence, cells were detached with trypsin–EDTA (trypsin–ethylenediaminetetraacetic acid) and seeded at the indicated densities. Cells between passages 15 and 35 were used for experiments.

### 3.8. Assessment of Cellular Viability

The CellTiter 96^®^ AQueous One Solution Cell Proliferation Assay (Promega, Madison, WI, USA) was used to evaluate the effect of WPC digests on cell viability. The CellTiter 96^®^ AQueous One Solution reagent contains the tetrazolium compound 3-(4,5-dimethylthiazol-2-yl)-5-(3-carboxymethoxyphenyl)-2-(4-sulfophenyl)-2H-tetrazolium, inner salt (MTS), and an electron-coupling reagent, phenazine ethosulfate (PES). Cells were seeded in 96-well plates at a density of 7 *×* 10^4^ cells/cm^2^ and incubated for 24 h. After 24 h, gastrointestinal (GI) digests at the indicated concentrations were added, and the cells were incubated for an additional 48 h. The assay was performed by adding 10 µL of the CellTiter 96^®^ AQueous One Solution reagent per well, incubating for 45 min, and then measuring absorbance at 490 nm using a BioTek Synergy HT microplate reader (Mason Technology, Dublin, Ireland). Measurements were performed in triplicate and results were expressed as percent cell viability relative to the untreated control (set to 100%).

### 3.9. CCK Secretion Study

Assays were performed as described Santos-Hernández et al. [12], with minor modifications. Cells were cultured in 24-well plates for 48 h at a density of 3 × 10^5^ cells per well. The cells were washed twice with HEPES buffer (20 mM HEPES, 10 mM glucose, 140 mM NaCl, 4.5 mM KCl, 1.2 mM CaCl_2_, 1.2 mM MgCl_2_, pH 7.4) prior to treatment with WPC digests, fractions or HEPES buffer (control). Digest fractions at three concentrations (1, 2, and 4 mg mL^−1^) were applied and cells were incubated for 2 h. Supernatants were collected and stored at −80 °C, supplemented with Halt Protease and Phosphatase Inhibitor Cocktail (Thermo Fisher Scientific, Waltham, MA, USA). CCK concentration was measured using a commercial CCK (26–33), nonsulfated enzyme immunoassay kit (Phoenix Pharmaceuticals Inc., Burlingame, CA, USA). Assays were performed according to the manufacturer’s instructions. Experiments were performed in triplicate, each with duplicate technical replicates.

### 3.10. Peptide Identification by Tandem Mass Spectrometry (HPLC–MS/MS)

The <3 kDa fraction obtained after 120 min of intestinal digestion of WPC, identified as the most active, was analyzed by HPLC–MS/MS. Lyophilized samples were reconstituted at 1 mg protein mL^–1^ in solvent A (water with 0.1% (*v*/*v*) formic acid), centrifuged at 13,000× *g* for 10 min, and the supernatant was filtered through a 0.22 µm µm membrane; the injection volume was 50 µL. Chromatographic separation was performed on an Agilent 1200 HPLC system (Agilent Technologies, Palo Alto, CA, USA) coupled to an Esquire–LC ion trap (Bruker Daltonics, Billerica, MA, USA) equipped with an electrospray ionization (ESI) source in positive-ion mode, with tandem MS acquired using collision-induced dissociation (CID), using an Agilent AdvanceBio C18 column (150 × 2.1 mm, 2.7 µm, 120 Å) at a flow rate of 0.2 mL/min and maintained at 40 °C. Nitrogen was used as the nebulizer and drying gas; source parameters were as follows: nebulizer pressure, 60 psi; drying gas flow, 8 L/min; and temperature, 350 °C. The capillary voltage was set to 4.5 kV, and helium was used as the damping gas at manufacturer-default settings. The MS/MS acquisition threshold was set to 10,000 counts. Peptides were eluted with a linear gradient of 0–45% solvent B (acetonitrile with 0.1% (*v*/*v*) formic acid) over 120 min, preceded by a 5 min pre-equilibration at 0% B, followed by a 5 min wash at 95% B and a 10 min re-equilibration at 0% B. The mass analyzer acquisition range was set to an m/z range of 200–2500. Spectral data (*m*/*z*) were processed individually using DataAnalysis (Bruker Daltonics, Billerica, MA, USA). Peptide identification and sequencing were performed with the Mascot search engine (version 2.6, Matrix Science) against an expanded UniProt database (taxonomy: Bos taurus) including specific whey protein entries (β-lactoglobulin, α-lactalbumin), a set of cRAP contaminants (common Repository of Adventitious Proteins), and a target–decoy strategy for FDR (false discovery rate) estimation. Quality-control criteria were applied, including an FDR of ≤ 1% at both the PSM (peptide–spectrum match) and peptide levels (decoy-based), a significance threshold of *p* < 0.05, and manual inspection of representative spectra.

### 3.11. In Silico Analysis of Peptide Bioactivity

The bioactivity potential of peptides identified by LC–MS/MS was evaluated using the MultiPep platform (https://agbg.shinyapps.io/MultiPep, accessed on 18 March 2025) [57], which applies machine-learning models trained on curated peptide datasets to predict multiple biological activities. MultiPep is a web-based, multi-label deep-learning approach designed to classify peptide bioactivities, to identify candidate bioactive peptides. Peptide sequences were submitted in FASTA format, and predictions were performed under default parameters. Each peptide received probability scores (0–1), with higher values indicating a stronger likelihood of bioactivity. For this study, peptides with MultiPep scores ≥ 0.70 were retained as candidate bioactive peptides for downstream interpretation. To benchmark predictions against experimentally reported motifs, sequences were also queried against the BIOPEP-UWM database (http://www.uwm.edu.pl/biochemia/index.php/en/biopep, accessed on 27 March 2025) to identify previously documented bioactive fragments and associated activities. Functional categories screened included antihypertensive, ACE-inhibitory, antidiabetic (including DPP-IV inhibition), antioxidant and anti-inflammatory. Peptide sketches and basic physicochemical descriptors (e.g., length, mass, net charge, and pI) were generated with PepDraw (https://www.pepdraw.com/, accessed on 16 November 2024), and data handling/visualization were performed in R (version 4.3.2; R Foundation for Statistical Computing, Vienna, Austria).

Overall, combining MultiPep prediction scores with BIOPEP-UWM annotations provided a dual strategy to prioritize novel whey-derived peptides with potential health-promoting activities while benchmarking against experimentally documented bioactive sequences.

### 3.12. Statistical Analysis

Before statistical analysis, normality and homoscedasticity were assessed using the Shapiro–Wilk and Levene’s tests (*p* > 0.05 was considered indicative of assumption compliance). For response variables that did not meet these assumptions, models were refit to rank-transformed data, as described by Conover & Iman [58]. Whey protein digestibility across time points (30, 60, 90, 120, 150, 180, 210, 240 min) was analyzed by one-way ANOVA, treating time (min) as a fixed factor with independent observations. The effect of in vitro gastrointestinal digestion on the free amino acid profile was analyzed by two-way ANOVA. Furthermore, the effects of whey protein digests and fractions with different concentrations on CCK secretion in STC-1 cells were evaluated by three-way ANOVA. Tukey’s honestly significant difference (HSD) post hoc test was used for multiple pairwise comparisons, with α = 0.05. Analyses were performed in R (R Core Team, Vienna, Austria) using RStudio (version 2023.12.1, Boston, MA, USA) and figures were generated in SigmaPlot 10.0 Systat Software Inc., San Jose, CA, USA) and WebLogo 2.8.2, University of California, Berkeley, CA, USA).

## 4. Conclusions

Simulated gastrointestinal digestion of whey protein concentrate generated extensive proteolysis, yielding a complex mixture of low-molecular-weight peptides and a marked increase in branched-chain and aromatic amino acids recognized as activators of nutrient-sensing receptors. Among the digested fractions, the <3 kDa intestinal fraction elicited the most pronounced and dose-dependent CCK response in STC-1 enteroendocrine cells, indicating that short-chain peptides, rather than intact proteins, are the main drivers of enteroendocrine activation under the conditions tested. Peptidomic analysis of this fraction identified 130 peptides derived primarily from β-lactoglobulin and α-lactalbumin. These peptides are short (5–13 residues), enriched in hydrophobic and aromatic residues, and include ENSAEPE-based neuropeptide-like sequences (ENSAEPEQS, ENSAEPEQSL, ENSAEPEQSLV, and MENSAEPEQSLV). Such structural features closely resemble those recently reported for CCK- and GLP-1–releasing peptides that act through CaSR- and GPCR-dependent mechanisms, providing a plausible structural framework linking whey-derived peptides to gut hormone secretion along the gut–brain axis. Of the 130 identified peptides, 70 displayed MultiPep scores ≥ 0.70 and were selected for in silico bioactivity profiling. This analysis revealed a high prevalence of candidates with predicted antihypertensive and ACE-inhibitory activities, as well as antidiabetic and DPP-IV–inhibitory functions, underscoring a multifunctional bioactivity landscape in which many CCK-related sequences may concurrently confer cardiometabolic benefits. To our knowledge, this is among the first studies to integrate MultiPep prediction with an experimentally derived whey peptidome in the context of gut hormone modulation. Overall, our results support digestion-derived whey peptides as multifunctional bioactive molecules that combine structural determinants compatible with CCK/GLP-1 stimulation and predicted antihypertensive/antidiabetic activities. These properties highlight their potential for use in nutritional strategies aimed at enhancing satiety, regulating appetite and energy intake, and improving metabolic health, as well as in the rational design of peptide-enriched functional foods and nutraceuticals. Targeted in vitro and in vivo studies, including receptor-level characterization and dose–response evaluation of individual peptides, are warranted to validate the most potent CCK-stimulatory candidates and further elucidate their mechanisms of action along the gut–brain axis.

## Figures and Tables

**Table 1:** Biological activities and physicochemical characteristics of peptides matching sequences found in α-lactalbumin (α-La) and β-lactoglobulin (β-Lg).

Amino Acid Sequence	Protein	Residue Range	Reported Bioactivity	Molecular Mass (Da)	Isoelectric Point (pI)
FHTSGYDTQA	α–La	30–40	DPP–IV Inhibitor	1125.4712	4.98
CKDDQNPH	α–La	61–68	Antibacterial	955.3806	5.14
DKVGINY	α–La	97–103	ACE inhibitor	807.4114	6.69
DKVGINYW	α–La	97–104	ACE inhibitor	993.4905	6.70
LAHKA	α–La	105–110	ACE inhibitor	538.3219	10.14
LCSEKLDQ	α–La	110–117	DPP–IV Inhibitor	934.4415	4.00
LIVTQTMK	β–Lg	1–8	Immunomodulator	932.5348	10.14
VLDTDY	β–Lg	94–99	ACE inhibitor	724.3268	2.88
TPEVDDEALEK	β–Lg	125–135	DPP–IV Inhibitor	1244.5752	3.43
LSFNPTQ	β–Lg	149–155	ACE inhibitor	805.3958	5.44

Theoretical monoisotopic mass and isoelectric point were obtained from the PepDraw.

## Data Availability

The original contributions presented in this study are included in the article and the Supplementary Materials. Further inquiries can be directed to the corresponding authors.

## Supplementary Materials

The following supporting information can be downloaded at https://www.mdpi.com/article/10.3390/molecules31020238/s1, Table S1: Two-way ANOVA results for the effect of in vitro gastrointestinal digestion on the free amino acid profile of whey protein, with digestion phase as a factor. Table S2: Two-way ANOVA results for the effect of whey protein digests on STC-1 cell viability, with concentration as a factor. Table S3: Sources of variation in the three-way ANOVA model for CCK secretion in STC-1 cells, comparing digestion phase, molecular-mass fraction, concentration, and interactions. Table S4: Mean, coefficient of variation (CV), and 95% confidence interval (CI) of CCK secretion for gastric and intestinal digests (<3 kDa and >3 kDa) at the three tested concentrations. Table S5: Peptides released from α-lactalbumin and β-lactoglobulin during simulated gastrointestinal digestion and identified by HPLC-MS/MS. Table S6: In silico bioactivity profiling (MultiPep) of α-lactalbumin- and β-lactoglobulin-derived peptides in the <3 kDa intestinal fraction. Figure S1. MS/MS spectra of whey-derived peptides detected in the <3 kDa intestinal fraction obtained after in vitro gastrointestinal digestion of whey protein concentrate.

## Abbreviations

The following abbreviations are used in this manuscript:

AA	Amino acids
AP	Protein content after in vitro digestion
ATCC	American Type Culture Collection
BP	Protein content before in vitro digestion
BSA	Bovine serum albumin
CaSR	Calcium-sensing receptor
CCK	Cholecystokinin
DMEM	Dulbecco’s Modified Eagle’s Medium
DPP-IV	Dipeptidyl peptidase-IV
EECs	Enteroendocrine cells
ELISA	Enzyme-linked immunosorbent assay
FBS	Fetal bovine serum
FDR	False discovery rate
GLP-1	Glucagon-like peptide-1
GPCR	G protein–coupled receptor
CID	Collision-induced dissociation
HPLC	High-performance liquid chromatography
LC–MS/MS	Liquid chromatography–tandem mass spectrometry
MWCO	Molecular weight cut-off
PBS	Phosphate-buffered saline
PYY	Peptide YY
SDS–PAGE	Sodium dodecyl sulfate–polyacrylamide gel electrophoresis
STC-1	Murine enteroendocrine cell line (ATCC CRL-3254)
WPC	Whey protein concentrate
α-La	α-lactalbumin
β-Lg	β-lactoglobulin
