# Supplementary material for: Release of Bioactive Peptides from Whey Protein During In Vitro Digestion and Their Effect on CCK Secretion in Enteroendocrine Cells: An In Silico and In Vitro Approach
Source: Molecules. 2026 Jan 10;31(2):238. doi: 10.3390/molecules31020238 (PMC12844283; doi:10.3390/molecules31020238)
Supplement: Supplementary file 1 [file molecules-31-00238-s001.zip › Supplementary_data-molecules.pdf]

Supplementary Materials

Table S1: Two–way ANOVA analysis values of effect of in vitro gastrointestinal digestion on amino acid profile (mg g^{-1} protein) of whey protein considering the effect of the Digestion phase.

	**df**	***F***	***p***
Digestion phase	1	12634	0.001
Amino acid	17	227	0.001
Digestion phase x Amino acid	17	34	0.001
Error	72		

The models were adjusted with the data transformed to "rank" since the response variables did not comply with the assumptions of normal error distribution and homogeneity of variances.

Table S2. Two–way ANOVA analysis values of effect of whey protein digests on the viability of the NIH/3T3 cell line considering the effect of concentration.

	**df**	***F***	***p***
Digestion phase	4	1.0374	0.41
Concentration	1	3.5313	0.07
Digestion phase x Concentration	4	1.3184	0.29
Error	20		

The models were adjusted with the data transformed to "rank" since the response variables did not comply with the assumptions of normal error distribution and homogeneity of variances.

Table S3. Source of variation of the three–way ANOVA model for CCK secretion in STC–1 cells comparing the three factors: Digestion phase, Fraction, and Concentration, and their respective interactions.

**Source of variation**	**df**	***F***	***p***	***Post hoc***
Digestion phase	1	1827.9	0.001	ID > GD
Fraction	2	1708.03	0.001	m > M
Concentration	2	571.82	0.001	4 > 2 > 1
Digestion phase x Fraction	2	245.89	0.001	
Digestion phase x Concentration	2	95.83	0.001	
Fraction x Concentration	2	2.85	NS	NS
Digestion phase x Fraction x Concentration	2	18.38	0.001	
Error	98			

NS: Not Significant, ID: Intestinal Digest, GD: Gastric Digest, m: < 3 kDa, M: > 3 kDa, 4: 4 mg mL^{-1}, 2: 2 mg mL^{-1}, 1: 1 mg mL^{-1}. The models were adjusted with the data transformed to "rank" since the response variables did not comply with the assumptions of normal error distribution and homogeneity of variances.

Table S4. Mean, coefficient of variation (CV), and 95% confidence interval (CI) of CCK secretion for gastric and intestinal digests (<3 kDa and >3 kDa) at the three evaluated concentrations.

Phase of digestion	Fraction	Concentration	Mean	CV	CI 95%
Gastric phase	<3 kDa	Control	21.04	12.90	2.27
		1 mg-mL^{-1}	146.35	4.80	6.96
		2 mg-mL^{-1}	174.85	6.64	9.71
		4 mg-mL^{-1}	240.20	5.02	10.07
	>3 kDa	Control	21.04	12.90	2.27
		1 mg-mL^{-1}	118.16	2.21	2.18
		2 mg-mL^{-1}	158.63	1.48	1.96
		4 mg-mL^{-1}	210.77	2.78	4.89
Intestinal phase	<3 kDa	Control	21.04	12.90	2.27
		1 mg-mL^{-1}	285.17	1.48	3.53
		2 mg-mL^{-1}	375.43	0.75	2.34
		4 mg-mL^{-1}	383.43	1.59	5.11
	>3 kDa	Control	21.04	12.90	2.27
		1 mg-mL^{-1}	193.14	2.40	3.88
		2 mg-mL^{-1}	218.99	2.42	4.43
		4 mg-mL^{-1}	238.15	1.55	3.09

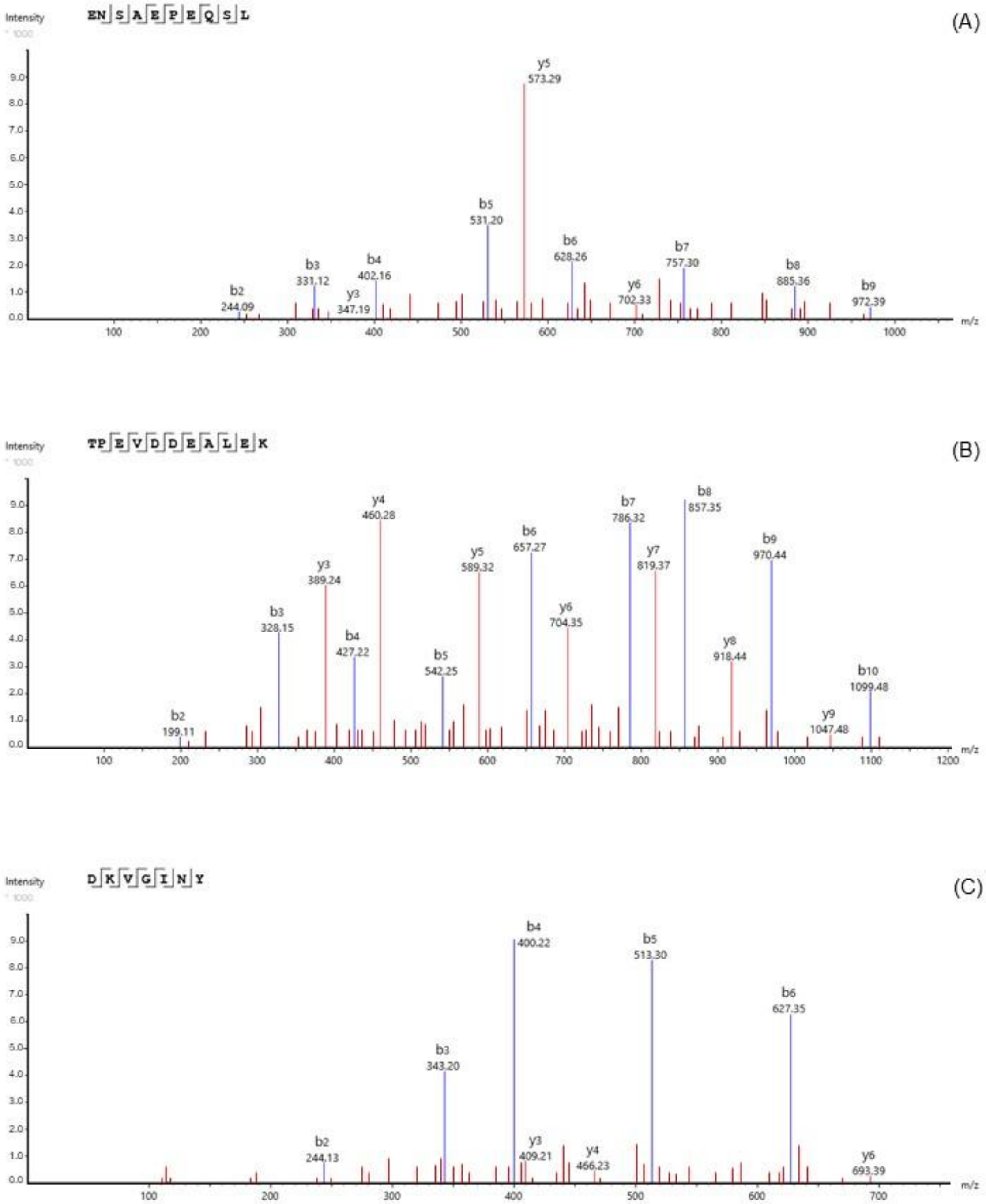

Figure S1. MS/MS spectra of whey-derived peptides detected in the <3 kDa intestinal fraction obtained after in vitro gastrointestinal digestion of whey protein concentrate. (A) ENSAEPEQSL from β-Lg f(108–117), (B) TPEVDDEALEK from β-Lg f(125–135), and (C) DKVGINYW from α-La, f(97–104). Precursor ions and major fragment ions are annotated, with product ions assigned to the b- and y-ion series.
