# Supplementary material for: Release of Bioactive Peptides from Whey Protein During In Vitro Digestion and Their Effect on CCK Secretion in Enteroendocrine Cells: An In Silico and In Vitro Approach
Source: Molecules. 2026 Jan 10;31(2):238. doi: 10.3390/molecules31020238 (PMC12844283; doi:10.3390/molecules31020238)
Supplement: Supplementary file 1 [file molecules-31-00238-s001.zip › Table S5.pdf]

Table S5. Peptides released from α-lactalbumin and β-lactoglobulin during simulated in vitro gastrointestinal digestion, identified by HPLC–MS/MS in the <3 kDa fraction collected after 120 min of the intestinal phase. Physicochemical properties were calculated using PepDraw.

Sequence	Protein	Residue range	Sequence length	Molecular mass (Da)	Isoelectric point	Net charge at pH 7	Hydrophobicity
TKCEV	α-La	4–8	5	578.3	6.16	-0.05	14.10
CEVFRELK	α-La	6–13	8	1022.5	6.34	-0.05	16.33
EVFRELK	α-La	7–13	7	919.5	6.91	0	16.35
VSLPE	α-La	21–25	5	543.3	3.09	-1	10.42
PEWVCTTFHTSG	α-La	24–35	12	1363.6	5.06	-0.95	12.08
VCTTFHTS	α-La	27–34	8	894.4	7.17	0.05	9.25
TFHTSGYDTQA	α-La	30–40	11	1226.5	4.98	-0.91	15.08
TSGYD	α-La	33–37	5	541.2	2.95	-1.01	12.69
YDTQA	α-La	36–40	5	596.2	3.13	-1.01	12.35
DTQAI	α-La	37–41	5	546.3	3.11	-1	11.96
TQAIVQNN	α-La	38–45	8	886.4	5.62	-0.01	10.31
NNDSTE	α-La	44–49	6	678.2	2.88	-2.02	17.58
NNDSTEY	α-La	44–50	7	841.3	2.92	-2.02	16.87
NDSTE	α-La	45–49	5	564.2	2.88	-2.02	16.73
NDSTEYG	α-La	45–51	7	784.3	3.00	-2.02	17.17
STEYGLFQ	α-La	47–54	8	943.4	3.12	-1.01	10.49
TEYGLFQI	α-La	48–55	8	969.5	3.20	-1.01	8.91
TEYGLFQIN	α-La	48–56	9	1083.5	3.11	-1.01	9.76
LFQINNK	α-La	52–58	7	875.5	10.15	1	9.09
CKDDQNPH	α-La	61–68	8	955.4	5.14	-0.95	22.05
DDQNPH	α-La	63–68	6	724.3	3.91	-1.9	19.27

Sequence	Protein	Residue range	Sequence length	Molecular mass (Da)	Isoelectric point	Net charge at pH 7	Hydrophobicity
NISCD	α-La	74–78	5	550.2	2.95	-1.07	11.71
NISCDK	α-La	74–79	6	678.3	6.09	-0.07	14.51
LDDDL	α-La	81–85	5	589.3	2.84	-3	16.32
LDDDLTD	α-La	81–87	7	805.3	2.61	-4	20.21
LDDDLTDD	α-La	81–88	8	920.1	2.56	-5	23.85
DLTDDIM	α-La	84–90	7	821.75	2.75	-3	16.03
TDDIM	α-La	86–90	5	593.2	2.85	-2.01	13.64
CVKKILDKVGIN	α-La	91–102	12	1328.8	10.11	1.95	17.51
KILDKVGINY	α-La	94–103	10	1161.7	9.41	0.99	14.48
DKVGINY	α-La	97–103	7	807.4	6.77	0	14.05
DKVGINYW	α-La	97–104	8	993.5	6.77	0	11.96
KVGIN	α-La	98–102	5	529.3	9.81	0.99	11.12
LAHKAL	α-La	105–110	6	651.4	10.15	1.1	11.53
AHKALCSEKLD	α-La	106–116	11	1213.6	7.17	0.05	22.04
KALCSEK	α-La	108–114	7	777.4	8.66	0.94	16.82
LCSEKLDQ	α-La	110–117	8	934.4	4.00	-1.05	16.68
LIVTQTMK	β-Lg	1–8	8	932.5	10.15	1	8.47
LIVTQTMKGL	β-Lg	1–10	10	1102.6	10.15	1	8.37
IVTQTMK	β-Lg	2–8	7	819.5	10.16	1	9.72
VTQTMK	β-Lg	3–8	6	706.4	10.15	1	10.84
TQTMKGLD	β-Lg	4–11	8	892.4	6.50	-0.01	14.84
KGLDI	β-Lg	8–12	5	544.3	6.48	-0.01	13.12
GLDIQ	β-Lg	9–13	5	544.3	3.04	-1	11.09
GLDIQKV	β-Lg	9–15	7	771.4	6.82	0	13.43

Sequence	Protein	Residue range	Sequence length	Molecular mass (Da)	Isoelectric point	Net charge at pH 7	Hydrophobicity
LDIQKV	β-Lg	10–15	6	714.4	6.80	0	12.28
LDIQKVA	β-Lg	10–16	7	785.5	6.80	0	12.78
DIQKVAG	β-Lg	11–17	7	729.4	6.87	0	15.18
YSLAMAA	β-Lg	20–26	7	725.3	5.76	-0.01	7.23
LAMAASDISL	β-Lg	22–31	10	990.5	3.12	-1	9.67
DISLLDA	β-Lg	28–34	7	745.4	2.95	-2	12.52
LLDAQSA	β-Lg	31–37	7	716.4	3.13	-1	11.27
LDAQSAP	β-Lg	32–38	7	700.3	2.93	-1	12.66
DAQSAPL	β-Lg	33–39	7	700.3	3.12	-1	12.66
DAQSAPLRV	β-Lg	33–41	9	955.5	6.91	0	14.01
AQSAP	β-Lg	34–38	5	472.2	5.91	0	10.27
AQSAPLRV	β-Lg	34–41	8	840.5	11.18	1	10.37
YVEELK	β-Lg	42–47	6	779.4	4.09	-1	15.54
YVEELKPTPE	β-Lg	42–51	10	1203.6	3.79	-2	19.7
VEELKP	β-Lg	43–48	6	713.4	4.08	-1	16.39
VEELKPT	β-Lg	43–49	7	814.4	4.08	-1	16.64
VEELKPTPE	β-Lg	43–51	9	1040.5	3.79	-2	20.41
EELKPT	β-Lg	44–49	6	715.4	4.08	-1	17.1
EELKPTP	β-Lg	44–50	7	812.4	4.08	-1	17.24
EELKPTPE	β-Lg	44–51	8	941.5	3.79	-2	20.87
ELKPT	β-Lg	45–49	5	586.3	6.76	0	13.47
ELKPTPE	β-Lg	45–51	7	812.4	4.08	-1	17.24
KPTPEG	β-Lg	47–52	6	627.3	6.57	-0.01	16.01
KPTPEGD	β-Lg	47–53	7	742.3	4.00	-1.01	19.65

Sequence	Protein	Residue range	Sequence length	Molecular mass (Da)	Isoelectric point	Net charge at pH 7	Hydrophobicity
KPTPEGDL	β-Lg	47–54	8	855.4	4.01	-1.01	18.4
KPTPEGDLE	β-Lg	47–55	9	984.5	3.73	-2.01	22.03
PTPEGDLEI	β-Lg	48–56	9	969.5	2.89	-3	18.11
PTPEGDLEIL	β-Lg	48–57	10	1082.5	2.90	-3	16.86
PTPEGDLEILLQK	β-Lg	48–60	13	1451.8	3.73	-2	19.18
PEGDLE	β-Lg	50–55	6	658.3	2.79	-3	18.84
EGDLE	β-Lg	51–55	5	561.2	2.79	-3	18.7
EGDLEI	β-Lg	51–56	6	674.3	2.89	-3	17.58
EGDLEILL	β-Lg	51–58	8	900.5	2.9	-3	15.08
GDLEILL	β-Lg	52–58	7	771.4	2.99	-2	11.45
GDLEILLQK	β-Lg	52–60	9	1027.6	4.00	-1	15.02
KWENGE	β-Lg	60–65	6	761.3	4.08	-1.01	17.87
KWENGEC	β-Lg	60–66	7	864.3	4.08	-1.05	17.85
WENGEC	β-Lg	61–66	6	736.2	2.84	-2.05	15.05
WENGECAQ	β-Lg	61–68	8	935.3	2.96	-2.05	16.32
WENGECAQK	β-Lg	61–69	9	1063.4	4.09	-1.05	19.12
ENGECAQ	β-Lg	62–68	7	749.3	2.96	-2.05	18.41
IAEKTK	β-Lg	72–77	6	688.4	9.94	1	16.76
IAEKTKI	β-Lg	72–78	7	801.5	9.94	1	15.64
AEKTKIPA	β-Lg	73–80	8	856.5	9.99	1	17.4
AEKTKIPAV	β-Lg	73–81	9	955.6	9.99	1	16.94
KTKIPA	β-Lg	75–80	6	656.4	10.57	1.99	13.27
KTKIPAV	β-Lg	75–81	7	755.5	10.57	1.99	12.81
LNENK	β-Lg	87–91	5	616.3	6.88	0	14.78

Sequence	Protein	Residue range	Sequence length	Molecular mass (Da)	Isoelectric point	Net charge at pH 7	Hydrophobicity
LNENKVLVL	β-Lg	87–95	9	1040.6	6.88	0	11.36
NENKVLV	β-Lg	88–94	7	814.5	6.4	-0.02	13.86
KVLVL	β-Lg	91–95	5	570.4	9.81	0.99	7.28
VLDTDY	β-Lg	94–99	6	724.3	2.88	-2	13.01
DTDYKK	β-Lg	96–101	6	768.4	6.89	0	20.32
MENSAEPEQSLV	β-Lg	107–118	12	1332.6	2.96	-3	19.59
ENSAEPE	β-Lg	108–114	7	774.3	2.83	-3	20.74
ENSAEPEQS	β-Lg	108–116	9	989.4	2.87	-3	21.97
ENSAEPEQSL	β-Lg	108–117	10	1102.5	2.93	-3	20.72
ENSAEPEQSLV	β-Lg	108–118	11	1201.5	2.96	-3	20.26
NSAEPEQS	β-Lg	109–116	8	860.3	2.97	-2.02	18.34
SAEPEQ	β-Lg	110–115	6	659.3	2.96	-2	17.03
SAEPEQS	β-Lg	110–116	7	746.3	2.97	-2	17.49
AEPEQS	β-Lg	111–116	6	659.3	2.97	-2	17.03
AEPEQSL	β-Lg	111–117	7	772.4	3.03	-2	15.78
PEQSLA	β-Lg	113–118	6	643.3	3.21	-1	12.15
QCLVRTPE	β-Lg	120–127	8	944.5	6.16	-0.05	12.77
CLVRTP	β-Lg	121–126	6	687.4	9.50	0.95	8.37
TPEVDD	β-Lg	125–130	6	674.3	2.71	-3.01	18.74
TPEVDDE	β-Lg	125–131	7	803.3	2.71	-4	22.37
TPEVDDEA	β-Lg	125–132	8	874.4	2.82	-4	22.87
TPEVDDEALE	β-Lg	125–134	10	1116.5	2.66	-5	25.25
TPEVDDEALEK	β-Lg	125–135	11	1244.6	3.43	-4	28.05
PEVDDEA	β-Lg	126–132	7	773.3	2.82	-4	22.62

Sequence	Protein	Residue range	Sequence length	Molecular mass (Da)	Isoelectric point	Net charge at pH 7	Hydrophobicity
PEVDDEAL	β-Lg	126–133	8	886.4	2.81	-4	21.37
PEVDDEALEK	β-Lg	126–135	10	1143.5	3.43	-4	27.8
EVDDEA	β-Lg	127–132	6	676.3	2.82	-4	22.48
EVDDEALEK	β-Lg	127–135	9	1046.5	3.43	-4	27.66
VDDEAL	β-Lg	128–133	6	660.3	2.87	-3	17.6
VDDEALEK	β-Lg	128–135	8	917.4	3.53	-3	24.03
DDEALEK	β-Lg	129–135	7	818.4	3.53	-3	24.49
DEALEK	β-Lg	130–135	6	703.3	3.73	-2	20.85
LSFNPTQ	β-Lg	149–155	7	805.8	5.96	0	7.41
LSFNPTQL	β-Lg	149–156	8	918.5	6.04	0	6.16
SFNPT	β-Lg	150–154	5	564.3	5.65	-0.01	7.89
LEEQCH	β-Lg	156–161	6	757.3	4.07	-1.95	16.99
EEQCH	β-Lg	157–161	5	644.2	4.07	-1.95	18.24
